# An insight into brown/beige adipose tissue whitening, a metabolic complication of obesity with the multifactorial origin

**DOI:** 10.3389/fendo.2023.1114767

**Published:** 2023-02-16

**Authors:** Khanyisani Ziqubu, Phiwayinkosi V. Dludla, Sinenhlanhla X. H. Mthembu, Bongani B. Nkambule, Sihle E. Mabhida, Babalwa U. Jack, Tawanda M. Nyambuya, Sithandiwe E. Mazibuko-Mbeje

**Affiliations:** ^1^ Department of Biochemistry, North-West University, Mmabatho, South Africa; ^2^ Biomedical Research and Innovation Platform, South African Medical Research Council, Tygerberg, South Africa; ^3^ Department of Biochemistry and Microbiology, Faculty of Science and Agriculture, University of Zululand, KwaDlangezwa, South Africa; ^4^ School of Laboratory Medicine and Medical Sciences, University of KwaZulu-Natal, Durban, South Africa; ^5^ Department of Health Sciences, Faculty of Health and Applied Sciences, Namibia University of Science and Technology, Windhoek, Namibia

**Keywords:** brown adipose tissue, beige adipose tissue, whitening, obesity, metabolic complications

## Abstract

Brown adipose tissue (BAT), a thermoregulatory organ known to promote energy expenditure, has been extensively studied as a potential avenue to combat obesity. Although BAT is the opposite of white adipose tissue (WAT) which is responsible for energy storage, BAT shares thermogenic capacity with beige adipose tissue that emerges from WAT depots. This is unsurprising as both BAT and beige adipose tissue display a huge difference from WAT in terms of their secretory profile and physiological role. In obesity, the content of BAT and beige adipose tissue declines as these tissues acquire the WAT characteristics *via* the process called “whitening”. This process has been rarely explored for its implication in obesity, whether it contributes to or exacerbates obesity. Emerging research has demonstrated that BAT/beige adipose tissue whitening is a sophisticated metabolic complication of obesity that is linked to multiple factors. The current review provides clarification on the influence of various factors such as diet, age, genetics, thermoneutrality, and chemical exposure on BAT/beige adipose tissue whitening. Moreover, the defects and mechanisms that underpin the whitening are described. Notably, the BAT/beige adipose tissue whitening can be marked by the accumulation of large unilocular lipid droplets, mitochondrial degeneration, and collapsed thermogenic capacity, by the virtue of mitochondrial dysfunction, devascularization, autophagy, and inflammation.

## 1 Introduction

Adipose tissue is an endocrine organ that has become the central focus in research toward a better understanding of the pathological mechanisms associated with obesity (1, 2). The latter is characterized by adipose tissue hypertrophy “adipocytes expansion” and hyperplasia “increase adipocytes number” caused by a chronic imbalance between energy intake and expenditure (1, 3). There are three distinct types of adipocytes that have been well studied in mammals (4): (i.) white adipocytes comprised of large single lipid droplet and few mitochondria, and this form of adipose is predominately useful to facilitate the storage of excess energy in a form of fats (5), and (ii.) brown adipocytes, and (iii.) beige or brite adipocytes rich in mitochondria, which are important organelles for regulating thermogenesis, mainly *via* the action of uncoupling protein 1 (UCP1) (5). Although brown and beige adipocytes share thermogenic capacity, several characteristics established that both are distinct cell types, which are different in terms of origin, anatomical location, and plasticity (6, 7). Of note, beige adipocytes emerge from white adipose tissue (WAT) depots *via* the process called “browning” (8, 9).

The (re)discovery of brown (BAT) and beige adipose tissue has garnered interest as therapeutic targets to promote energy expenditure and counteract complications linked with obesity (7). Notably, previous conversations have emphasized the importance of sufficient vasculature to improve mitochondrial function in BAT and alleviate obesity-associated complications (10). The vascular rarefaction in BAT was associated with the dysfunction and loss of BAT commonly referred to as “whitening” (11). It is well accepted that both BAT and beige adipose tissue are subjected to a whitening effect which is common in obesity, whereby they acquire unilocular cells that gradually lose all the brown characteristics and assume WAT characteristics (12). Moreover, the whitening of adipose tissue is accompanied by lipid accumulation due to reduced substrate oxidation and the loss of mitochondria through the impairment of molecular mechanisms regulating thermogenesis, as well as those involving autophagy and mitophagy (12). Others have demonstrated that brown-to-white adipose tissue conversion may activate undesirable metabolic complications such as inflammatory response and, the much-explored pyrin domain-containing protein 3 (NLRP3) inflammasome (13). Recently, BAT whitening was defined as a long-term obesity complication that displayed a progressive severity upon chronic intake of a high-fat diet (HFD) during the pathogenesis of obesity (14). Thus, with the rapidly rising prevalence of obesity (15), there is an urgent need to understand the causative and underlying factors implicated in BAT/beige adipose tissue whitening, including elucidating the molecular drivers of their plasticity. This encompasses deciphering pathophysiological mechanisms that implicate the development and aggravation of obesity, as shown by the fact that dysfunctional adipose tissue in obesity leads to a variety of secondary metabolic complications.

The current review elaborates on the prominent mechanisms involved in BAT/Beige adipose tissue whitening, while a sharp focus is placed on the impact of critical factors that contribute to obesity, such as diet, age, temperature, and various chemical substances. Importantly, an overview of BAT and beige adipose tissue, and their physiological relevance is given to highlight vital mechanisms implicated in the process of thermogenesis.

## 2 An overview of BAT and beige adipose tissue, and their physiological importance

**Figure 1:**
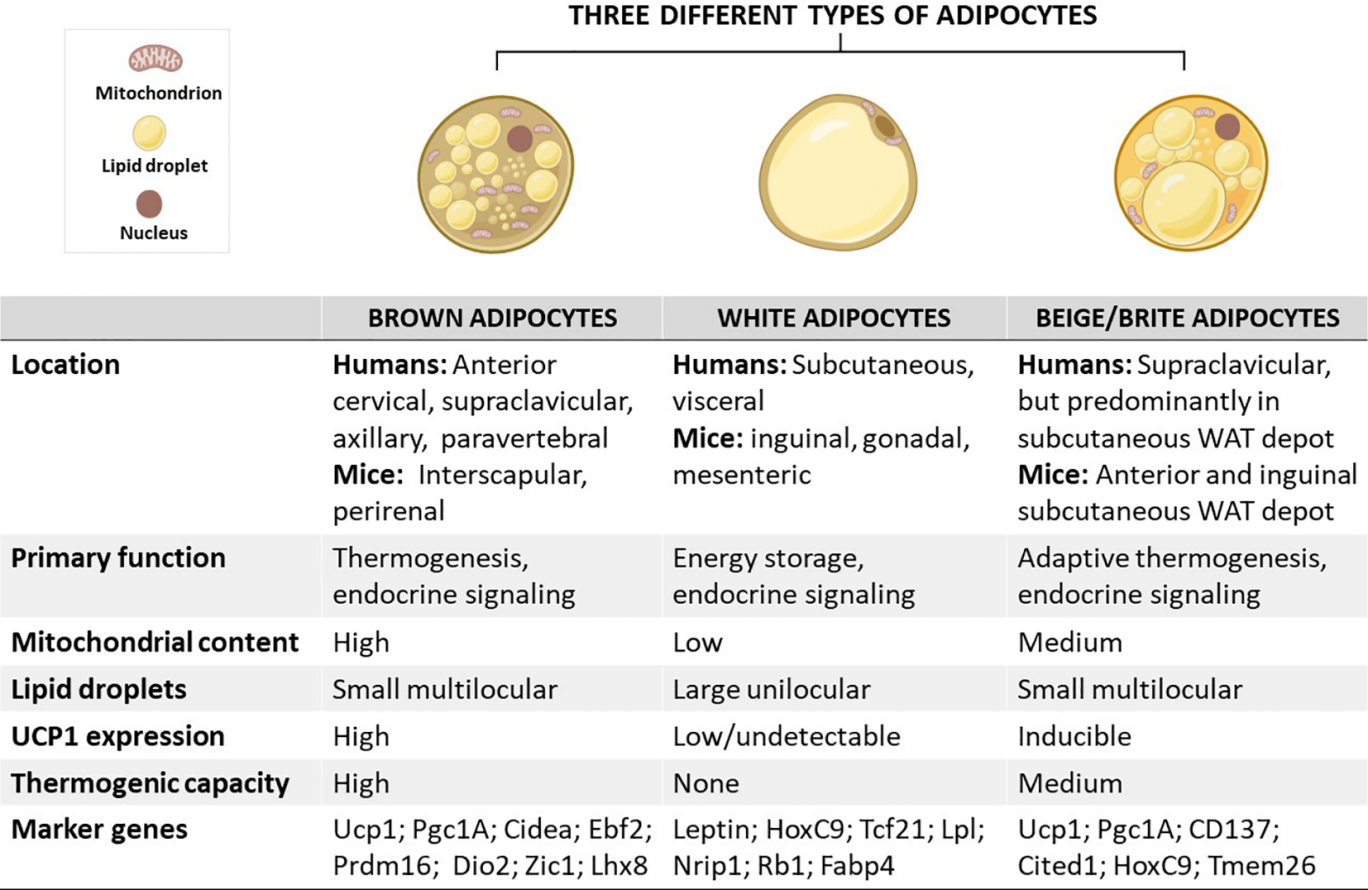
Schematic overview showing the characteristics of brown, white, and beige/brite adipocytes, including their localization, morphology, physiological function, and marker genes. In brief, the anatomical location of these adipocytes differs, except for white and beige adipocytes which are found within WAT depots. In terms of morphology and function, brown adipocytes are comprised of small multilocular lipid droplets, high mitochondrial content, and increased UCP1 expression, which can be also induced in beige adipocytes to promote thermogenesis and enhance energy expenditure, features that are important for the treatment of obesity. In sharp contrast, white adipocytes have fewer mitochondria and large unilocular lipid droplets to facilitate the storage of energy in a form of fats. Of note, these adipocytes have similar and distinct marker genes which could play an important role in tracking metabolic complications linked with obesity. Cidea, cell death-inducing DNA fragmentation factor-like effector A; CD137, tumour necrosis factor receptor superfamily, member 9; Dio2, Iodothyronine deiodinase 2; Lhx8, LIM homeobox protein 8; Pgc1A, peroxisome proliferator-activated receptor coactivator 1 alpha; Prdm16, PR domain-containing 16; Tcf21, Transcription factor 21; TMEM26, transmembrane protein 26; UCP1, uncoupling protein 1; WAT, white adipose tissue; Rb1, RB transcriptional corepressor 1; Zic1, zinc finger protein of the cerebellum.

As aforementioned, adipocytes are broadly classified into three distinct types: (i) brown adipocytes, (ii) white adipocytes, and (iii) beige or brite adipocytes (**Figure 1**) (4). These cells differ in terms of function and morphology, and their localization on various fat depots in mice and humans (16, 17). Importantly, brown adipocytes are enriched in the BAT depot, whereas both white and beige adipocytes are found within WAT depots (18). Predominantly, beige adipocytes emerge in subcutaneous WAT depots, including the anterior and inguinal subcutaneous WAT in mice (18, 19). In contrast to WAT, BAT was mainly viewed as the key site for upholding thermal homeostasis during cold adaptation in human infants (20). Despite a century of studies on neonatal BAT, the knowledge about BAT physiological features and the mechanisms by which this tissue regulates body temperature homeostasis in human neonates is scanty, mainly due to the lack of appropriate methods for such investigations. Recently, studies have utilized infrared thermography as a suitable non-invasive technique to investigate neonatal BAT activity (21, 22). It was reported that a single short-term cold exposure during the first day of life improves body temperature adaptation (21). The later rediscovered BAT and recruitable beige adipose tissue in adult humans which was initially thought to exist in newborn babies and hibernating animals only have highlighted the potential influence of this tissue in improving human health (23). This is attributable to the thermogenic and endocrine functions of these tissues to promote energy expenditure and secrete metabolic regulating molecules, called batokines, such as vascular endothelial growth factors (VEGF)-A, bone morphogenic protein 8b (BMP8b), neuregulin 4 (NRG4) and fibroblast growth factor 21 (FGF21) (24). Recent research has indicated that disease progression is consistent with impairment of gene expression levels of batokines regulating sympathetic neurite outgrowth, vascularization and glucose metabolism in animal model of T2D (25). Thus, the physiological functions of BAT and beige adipose tissue have spurred a new wave of interest in their effects on modulating metabolism. This is supported by mounting research on the health benefits of BAT, especially in combating obesity (26–28). Owing to this, several selective markers of adipose tissues have been identified and characterized as potential biomarkers in obesity, as reviewed by Pilkington et al. (29).

### 2.1 The development of BAT/beige adipose tissue: Implications in the pathogenesis of obesity

**Figure 2:**
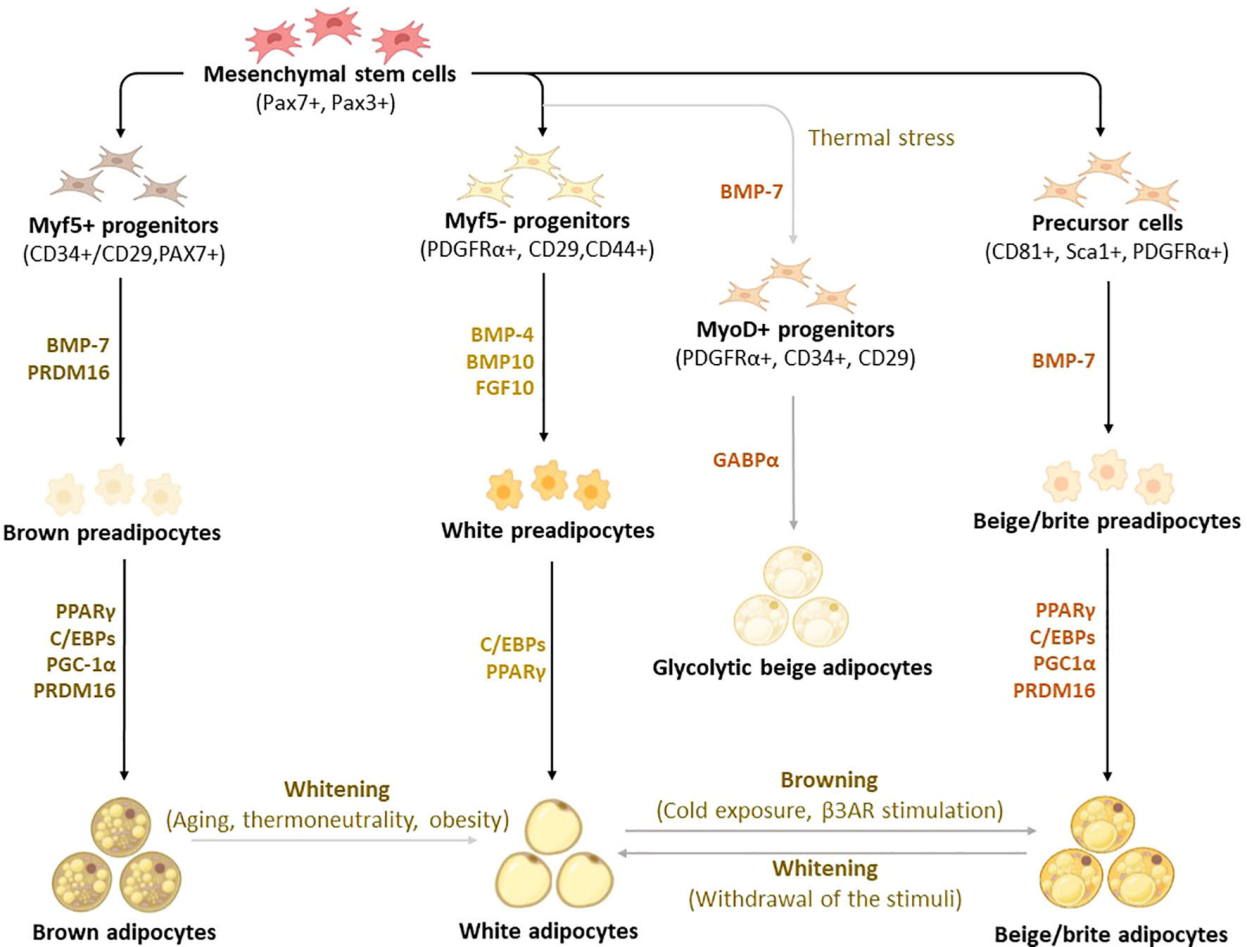
Differentiation and transdifferentiation trajectories of major adipocyte populations, including brown and white adipocytes, as well as canonical and glycolytic beige/brite adipocytes. Generally, brown adipocytes are derived from Myf5+ progenitors, whereas white and beige adipocytes originate from Myf5- progenitors that descend from mesenchymal stem cells. New advances on adipocyte origin show that there is another subpopulation of beige adipocytes known as glycolytic beige adipocytes, which are derived from MyoD+ progenitors within the stromal vascular fraction of inguinal WAT in response to thermal stress. During differentiation secretory factors BMP-7 and transcriptional factor PRDM16 induce Myf5+ cell commitment to brown preadipocytes whereas BMP-4, BMP-10, and FGF10 induce Myf5- cell commitment to white preadipocytes. On the other hand, BMP-7 induces a commitment of beige adipocyte precursors to canonical beige preadipocytes. Differentiation of all types of preadipocytes into mature adipocytes is driven by PPARγ, and C/EBPs, whereas brown and beige adipocytes require the expression of additional factors, including PRDM16 and PGC1α. Moreover, GABPα regulates the differentiation of glycolytic beige adipocytes. During transdifferentiation, white adipocytes convert to beige adipocytes following cold exposure or β3-AR stimulation. Likewise, brown and beige adipocytes can transdifferentiate into white-like adipocytes, a process termed whitening. β3-AR, beta-3 adrenergic receptor; BMP, bone morphogenetic protein; C/EBPs, CCAAT/enhancer-binding proteins; GABPα, GA-binding protein alpha; PGC1α, peroxisome proliferator-activated receptor coactivator 1 alpha; PPARγ, peroxisome proliferator-activated receptor-gamma; PRDM16, PR domain-containing 16; UCP1, Uncoupling protein 1.

Although brown, white, and beige adipocytes are derived from similar mesenchymal stem cells, they originate from different precursor cells with unique marker genes, as shown in **Figure 2** (30–33). Genetic-lineage tracing indicates that white adipocytes descend from Myf5- (PDGFRα+, CD29, CD44+) progenitors of mesenchymal stem cells (34, 35), while brown adipocytes originate from Myf5+ (CD34+/CD29, MYF5-, PAX3+) progenitors which differentiate into mature brown adipocytes (36–38). Interestingly, the existence of beige adipocytes which resemble white adipocytes in having a low basal expression of UCP1, but like classical brown adipocytes respond to thermogenic stimuli with high UCP1 expression (39). These cells are distinct types of thermogenic fat cells that originate from two distinct processes, *de novo* differentiation from Myf5- progenitor cells and transdifferentiation from white adipocytes *via* a process called “browning” (4). A study by Oguri et al. (40) showed that CD81+, Sca1+, PDGFRα+ adipocyte progenitors give rise to beige adipocytes and CD81 loss causes obesity, insulin resistance, and inflammation in mice. In addition to canonical beige adipocytes, a study of adipocyte heterogeneity has demonstrated that there is a subpopulation of thermal stress-induced glycolytic beige adipocytes that emerge from inguinal WAT (41, 42). Although cells in the MyoD1+ lineage do not typically give rise to any adipocytes, it was reported that glycolytic beige adipocytes descend from MyoD1+ progenitors that emerge within a stromal vascular fraction of inguinal WAT when beta-3 adrenergic receptor (β-AR) signaling is inhibited (41, 42). Thus, this has reignited the interest in studying and understanding the role of WAT browning in obesity (26, 28, 43). While white adipocytes transdifferentiate into beige adipocytes, both brown and beige adipocytes can transdifferentiate into white adipocytes through a process called “whitening”, which is quite common in obesity (44, 45). By now, it is evident that the differentiation process constitutes an essential component for understanding the developmental fate and function of BAT, which explains the increased exploration of these transcriptional factors in preclinical models of obesity (46–48).

Of note, bone morphogenetic protein (BMP)-7, a secretory protein that acts as an autocrine/paracrine mediator, promotes the differentiation of both brown and beige adipocytes in mice (49, 50). Recently, Townsend et al. (51) demonstrated that BMP7-loaded silk hydrogels into the subcutaneous WAT of mice induced brown adipogenesis in committed and uncommitted progenitor cells, which in turn increased energy expenditure and reduced weight gain in mice. In human-neck adipose-derived stromal cells, BMP7 enhanced mitochondrial DNA content, concomitant with increased gene expression of proliferator-activated receptor coactivator 1 alpha (PGC1α), a transcriptional co-regulator, responsible for mitochondrial biogenesis (52). Amongst other transcriptional factors, PR domain containing 16 (PRDM16) plays a key role in determining the fate of brown adipocyte differentiation (53). An elegant study by Seale et al. (54) has demonstrated that the loss of PRDM16 in brown adipose precursors results in the loss of brown adipocyte characteristics, which in turn promotes skeletal muscle differentiation instead of brown adipocyte differentiation. Importantly, PRDM16 is not only required for determining brown adipocyte fate but also for regulating thermogenic programming and maintenance of brown adipocyte identity (55). Adipogenic transcriptional factors, such as CCAAT enhancer-binding proteins (C/EBPs), and peroxisome proliferator-activated receptor (PPAR)-γ have long been known to play a central role in regulating adipocyte differentiation in almost all types of adipocytes (56–58). To further highlight the significance of studying adipocyte differentiation in obesity, a recently published protocol (59) compares white, beige, and brown adipocyte differentiation, further characterizing the expression of distinct transcriptional factors that are involved in thermogenesis. In fact, findings from this study indicate that differentiated pre-adipocytes from interscapular BAT has a higher thermogenic potential and expression levels of UCP1 with compared to WAT-derived cells. A comprehensive characterization of mature brown adipocyte subpopulations using single-nucleus ribonucleic acid (RNA) sequencing identified a rare subpopulation of adipocytes that increases in abundance at higher temperatures, suggesting a lower thermogenic activity (60). This subpopulation regulates the activity of neighboring adipocytes *via* acetate-mediated modulation of their thermogenic capacity (60).

### 2.2 Physiological functions of BAT/beige adipose tissue and their role in regulating metabolic health

#### 2.2.1 Activation of thermogenesis and the effect of various stimuli

**Figure 3:**
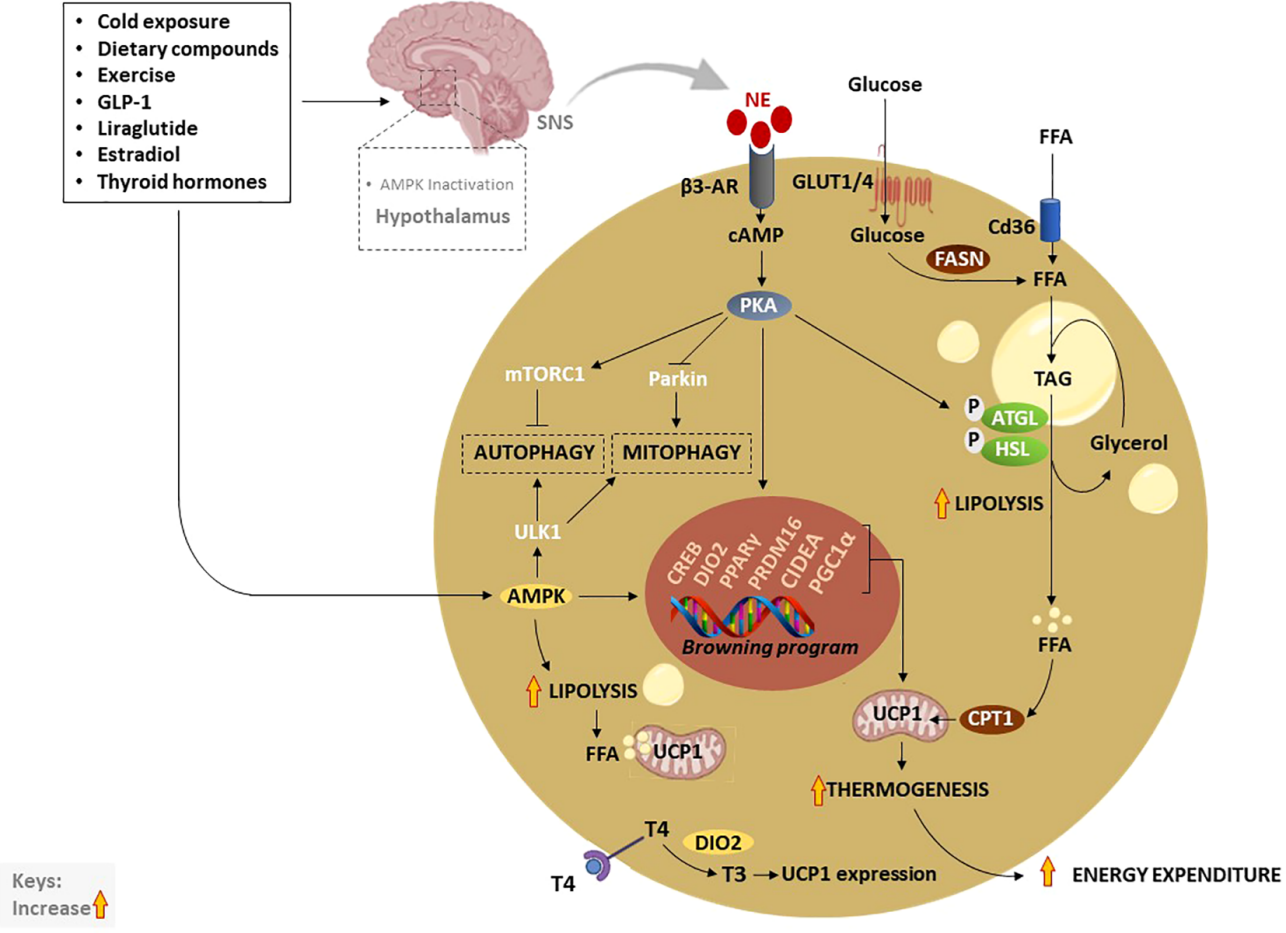
Thermogenic activation of brown and beige adipocytes by various exogeneous and endogenous stimuli. Traditionally, cold exposure and other dietary compounds activate thermogenesis *via* NE, excreted by the SNS, or through direct activation of AMPK. Inactivation of hypothalamic AMPK by thermogenic stimuli like estradiol, GLP-1, liraglutide or thyroid hormones, stimulate SNS resulting in the release of NE and stimulation of β3-AR which subsequently increase cAMP and activate PKA. Moreover, elevated cAMP activates CREB causing the transcription of thermogenic genes such as PPARγ, DIO2, PRDM16, PGC1α, and CIDEA. On the other hand, the cAMP-PKA signaling pathway promotes lipolysis just like AMPK through the phosphorylation of ATGL and HSL. Liberated FFA contributes to the upregulation of UCP1 and increases thermogenesis. In addition, enhanced TAG-derived fatty acids uptake upregulates CPT1, resulting in increased FFA transport into mitochondria. Glucose act as a substrate to generate FFA *via* FSAN, which ultimately contributes to heat production. Upon β3-AR stimulation, adenylate cyclase cAMP/PKA also activates mTORC1 and inhibits Parkin to repress autophagy/mitophagy, as a means to maintain beige adipocytes. Inversely, AMPK promotes promote autophagy/mitophagy to maintain mitochondrial health *via* the phosphorylating of ULK1 in brown adipocytes. AMPK, AMP-activated protein kinase; β3-AR, Beta-3 adrenergic receptor; PPARγ, peroxisome proliferator-activated receptor-gamma; CIDEA, cell death-inducing DNA fragmentation factor-like effector A; C/EBPs, CCAAT/enhancer-binding proteins; cAMP, cyclic adenosine monophosphate-protein kinase A; DIO2, Iodothyronine deiodinase 2; FASN, fatty acid synthase; FFA, free fatty acids; GLUTs, glucose transporters; HSL, hormone-sensitive lipase; NE, norepinephrine; PKA, protein kinase A; PRDM16, PR domain-containing 16; PGC1α, peroxisome proliferator-activated receptor coactivator 1 alpha; UCP1, Uncoupling protein 1; SNS, sympathetic nervous system; T4, thyroxine; T3, triiodothyronine; TAG, triacylglyceride.

A consistently growing body of literature indicates that elevating the thermogenic capacity of BAT through activation, recruitment, or BAT transplant could be an ideal approach to mitigate obesity (**Figure 3**) (61–63). The thermogenesis in BAT and beige adipose tissue is facilitated by UCP1 or thermogenin, a mitochondrial inner membrane protein that uncouples substrate oxidation from ATP synthesis, thereby dissipating excess energy as heat (64). In addition to a well-recognized UCP1-dependent thermogenic mechanism, there is a newly identified UCP1-independent thermogenic mechanism that could potentially offer a new target for the treatment of obesity and type 2 diabetes, especially targeting UCP1-negative adipocytes in the elderly and people with obesity (65–67). The UCP1-independent thermogenic mechanism involves ATP-dependent Calcium (Ca2+) cycling by Sarco/endoplasmic reticulum Ca2+-ATPase 2b, which enhances energy expenditure and glucose oxidation in beige adipocytes (68). A study by Okamatsu-Ogura et al. (68) has discovered that cold exposure induces UCP1-independent active lipid metabolism in BAT of UCP1-knockout mice. Recently, Oeckl et al. (69) identified the futile ATP-consuming triglyceride/fatty acid cycle as a central process that contributes to thermogenesis in BAT of UCP1-knockout mice. To date, UCP1 is the most investigated mitochondrial carrier protein, involved in the modulation of oxidative phosphorylation, with its isoforms like UCP2 also found in other tissues like the heart where they protect against oxidative stress (70). By now, it is well-accepted that cold exposure is a classical stimulus that utilizes glucose and fatty acids as substrates for adaptive thermogenesis (71). In humans, cold exposure increases glucose uptake and non-esterified fatty acid turnover, suggesting that activation of thermogenesis can help to improve plasma glucose clearance (71). Apart from the cold exposure, thermogenic stimuli, such as dietary compounds, physical exercise, and other various agents, including glucagon-like peptide-1 (GLP-1), thyroid hormones (THs), liraglutide and thiazolidinediones, are known to upregulate the genes involved in thermogenesis and induce WAT browning (26).

In general, thermogenesis is modulated by stimulating the sympathetic nervous system (SNS), which is in part regulated through the release of norepinephrine (NE) to activate β3-AR signaling in response to various stimuli (72). Amongst all other well-known mechanisms that regulate thermogenesis, the cyclic adenosine monophosphate-protein kinase A (cAMP-PKA) signaling pathway, and AMP-activated protein kinase (AMPK) are classical mechanisms that have been studied in depth (72). Upon activation, PKA phosphorylates adipose triglyceride lipase (ATGL) and hormone-sensitive lipase (HSL), enzymes that promote lipolysis, leading to the breakdown of triacylglyceride (TAG) to free fatty acids (FFA), again *via* AMPK (72–74). Resultant FFA can be further broken down and oxidized in the mitochondria by carnitine palmitoyltransferase 1 (CPT1) to produce heat *via* UCP1 (75). Of note, glucose transporter (GLUT)-1 and GLUT4 are expressed in human BAT to facilitate glucose uptake by brown adipocytes (76, 77). In response to chronic cold-exposure, glucose uptake by BAT is utilized for the fatty acid synthesis and β-oxidation process (78). Alternatively, cAMP-PKA signaling modulates the phosphorylation of cAMP-response element binding protein (CREB), which in turn promotes the transcription of mitochondrial biogenic and thermogenic genes, such as PGC1α, PRDM16, PPARγ, and UCP1 (79). Lipid-droplet-associated protein also known as cell death-inducing DNA fragmentation factor-like effector A (CIDEA) regulates UCP1 transcription for browning and thermogenesis in human adipocytes (80). In specific, CIDEA inhibits liver X receptor (LXR), a repression of UCP1 enhancer activity, and strengthens the binding of PPARγ to UCP1 enhancer, promoting UCP1 transcription in CIDEA knockout primary human adipocytes (80). Another prominent mechanism that is modulated by the kinase enzymes, PKA and AMPK is the regulation of autophagy “degradation of cellular components” or mitophagy “selective degradation of mitochondria” which plays an important role in BAT/beige adipose tissue plasticity (12, 81, 82). In BAT, activation of thermogenesis is paralleled by a reduction in the autophagic degradative activity, which converges at PKA that activates the rapamycin complex 1 (mTORC1) and inhibits Parkin transcription (83, 84). Moreover, melanocyte-inducing transcription factor (MITF) and forkhead box O3 (FOXO3) were identified as downstream autophagy-related transcription factors that are downregulated by cAMP-PKA during autophagy regression in beige adipocytes (83).

Inversely, AMPK is required for the efficient removal of damaged mitochondria or mitophagy through phosphorylation of unc-51-like kinase 1 (ULK1) as a means to maintain BAT and induce browning (85). Thus, targeting AMPK might be a plausible approach for the treatment of various metabolic diseases as recently reviewed by López (86). Dietary components such as polyphenols have been reported to activate the SNS and stimulate the release of THs, which in turn increase thermogenesis and induce WAT browning *via* the AMPK-PGC1α/Sirt1 and PPAR signaling pathways (87, 88). Apart from that, dietary intake alone is known to induce thermogenesis by increasing postprandial energy expenditure, contributing 5–15% of total daily energy expenditure (89). Other data has uncovered that WAT browning is a highly dynamic physiological process, in which O-linked b-N-acetylglucosamine (O-GlcNAc) signaling in agouti-related protein (AgRP) neurons is essential for suppressing thermogenesis to conserve energy upon fasting (90). In terms of hormonal control, both insulin and leptin act on hypothalamic or proopiomelanocortin neurons to promote WAT browning (91). Interestingly, overexpression of glucose-regulated protein 78 kDa/binding immunoglobulin protein (GRP78) in the ventromedial nucleus of the hypothalamus (VMH) alleviated ER stress and obesity; however, this was not dependent on leptin but it was related to the increased thermogenic activation of BAT and WAT browning (92). GLP-1, a hormone that controls satiation and glucose metabolism *via* vagal afferent neurons was reported to stimulate BAT thermogenesis by regulating sirtuin 1 (SIRT1) (93), and hypothalamic AMPK (94). Metabolism-activating hormones thyroxine (T4) and triiodothyronine (T3) which are mainly secreted by the thyroid gland convert from T4 to T3 by type II iodothyronine deiodinase (DIO2) promoting UCP1 expression in BAT (95). Importantly, THs target the hypothalamus in the brain to modulate energy balance *via* AMPK (96, 97). Specifically, THs act on the VMH to regulate the thermogenic program in BAT (98, 99). For instance, it was reported that the central T3 promotes hepatic lipogenesis and thermogenic program in BAT through the parasympathetic nervous system and SNS, respectively (98). These effects were coordinated by distinct signaling pathways in the VMH, JNK1, and ceramides/endoplasmic reticulum stress under the control of AMPK (98). Other fundamental hormones include estradiol, a female reproductive hormone, which inhibits AMPK leading to the activation of BAT thermogenesis *via* estrogen receptor alpha selectively in the VMH (100).

#### 2.2.2 The role of autophagy and inflammation on BAT/beige adipose tissue plasticity

Autophagy is a fundamental lysosomal catabolic process that degrades and recycles damaged cellular components such as lipids, proteins, and organelles for cellular survival in stress conditions (101–103). Although selective autophagic degradation of mitochondria termed “mitophagy” seems to be beneficial to eliminate damaged mitochondria from accumulated reactive oxygen species (ROS) in active BAT, there are some conflicting results in the literature regarding the importance of this process during the activation of BAT by cold exposure (84, 104–107). For example, Cairó et al. (107) reported that cold-induced thermogenic activation of BAT in mice was linked with autophagy repression, in part by upregulating cAMP-PKA pathway in NE-exposed brown adipocytes. Surprisingly, chronic cold exposure promoted autophagy/mitophagy in primary brown adipocytes and BAT from mice (105, 106). Yau et al. (106) showed similar effect on the activation of autophagy in BAT from mice in response to cold exposure, and this was marked by increased mRNA expression of the autophagic genes, including, sequestosome 1 *(Sqstm1*), autophagy-related (*Atg*)-5, *Atg7*, and *Ulk1*. This effect promoted β-oxidation, mitochondrial turnover, and thermogenesis in BAT (106). These results further indicate that autophagic processes in BAT are tightly regulated in response to cold exposure, as increasingly discussed (12, 83, 84).

Another hypothesis implementing the role of mitophagy on BAT/beige adipose tissue plasticity also prevails, and it is linked to the process of adipose tissue whitening (13, 83, 108, 109). For example, thermoneutrality reacclimatization after chronic cold exposure or withdrawal of thermogenic stimuli activate autophagic response and promotes the transition of BAT/beige adipose tissue to white-like adipose tissue in mice (83). Obviously, there are some distinct differences between BAT and beige adipose tissue whitening, such as that the brown adipocytes “disappear” from WAT depots and acquire unilocular phenotype with loss of UCP1 expression while brown adipocytes display an increased lipid accumulation, followed by protein degradation, and loss of protein synthesis without disappearance (12). Alternatively, reversal of beige-to-white adipocytes is tightly coupled with autophagy-mediated mitochondrial clearance after withdrawal of the external cues in mice (83). Notably, beige adipocytes progressively lose their morphological structure and molecular characteristics to acquire white-like characteristics bypassing an intermediate precursor stage (83). Inversely, inhibition of autophagy by UCP1+ adipocyte-specific deletion of Atg5 or Atg12 maintained functional beige adipocytes even after stimuli withdrawal (83). More evidence showed that inhibition of Parkin-mediated mitophagy underlies the maintenance of beige adipocyte in mice (108). Interestingly, physiological conditions such as aging can promote autophagy, which result to the loss of BAT activity. Indeed, upregulation of autophagy in BAT of mice is consistent with age-dependent decline of BAT activity and reduced metabolic rate (109). However, inhibition of mitophagy by BAT-specific deletion of the *Atg7* gene could improve insulin sensitivity and energy metabolism, as well as maintained higher mitochondrial content by suppressing mitochondrial clearance (109).

Beyond dysregulation of autophagy, inflammation is another instrumental process that may negatively influence BAT activity and compromise the metabolic rate of this tissue. Although the relationship between autophagy and inflammation is relatively complex, they both constitute a natural response to stress conditions. Apparently, elevated inflammatory status impairs brown adipocyte metabolic activity and promotes whitening (110). Kotzbeck et al. (13) showed that BAT whitening is coupled with a decreased mitochondrial content as whitened adipocytes become dysfunctional as they render low-grade inflammatory state that eventually leads to cell death. To further highlight the link between dysregulated autophagy and inflammation, it was demonstrated that activation of the NLRP3 inflammasome and increased expression of inflammatory markers, including F4/80, tumor necrosis factor-alpha (TNF-α), interleukin (IL)-1β, IL-10, IL-18, monocyte chemoattractant protein 1 (Mcp-1), and caspase-1 was linked with Atgl-deficiency in mice (13). Other studies showed that deletion of transcription factor nuclear factor erythroid-2, like-1 (Nfe2l1) induced ER stress, inflammation, mitochondrial dysfunction, insulin resistance, and whitening in BAT (111, 112). Altogether, mitophagic maintenance of the healthy network of mitochondria in BAT and beige adipose tissue is crucial for cell survival but requires a balanced remodeling system of mitochondrial biogenesis and degradation.

## 3 Factors inducing BAT/beige adipose tissue whitening or inactivation in obesity

### 3.1 Natural factors inducing BAT/beige adipose tissue whitening

While the browning of WAT has long been a growing area of interest in obesity research, whitening of BAT as an obesity-related complication with metabolic and health implications has been receiving less attention. Latterly, there is cumulative emerging evidence on BAT whitening, and it demonstrates that this phenomenon is multifactorial in origin. Notably, it has long been established that risk factors such as diet, age, genetics, as well as some chemicals can negatively influence the health of the general population (113). Risk factors such as excessive intake of an unhealthy diet can directly contribute to adiposity or weight gain (114), whereas aging is an undeniable major consequence that has long been linked with deteriorated health (115). Although acknowledged to significantly affect metabolic health, precise pathological mechanisms involved in this process are not completely understood. Interestingly, experimental evidence indicates that factors such as diet, thermoneutrality, age, and genetics can induce the whitening of BAT, and exacerbate obesity (**Table 1**).

**Table 1:** Summary of evidence reporting on BAT/beige adipose tissue whitening-induced by diet, thermoneutrality, age, and genetics.

Factors	Model	Intervention protocol	Main findings		Author, year
			Effect	Mechanism	
Diet	CD-1 mice	Mice fed HFD for 10 weeks	Increased fat deposition in BAT without altering triglyceride and free fatty acids levels the in blood	Not investigated	Gao et al., 2015 (116)
	C57BL/6 mice	Mice fed HFD for 10 weeks	Induced expansion of beige adipocytes residing in the WAT depot	Downregulated UCP1 gene expression	Dobner et al., 2017 (117)
	C57BL/6J mice	Mice fed HFD for 1, 3, or 7 day(s)	Increased lipid accumulation and insulin resistance in BAT	Upregulated Cd36, Hsl, Srebp1c, and downregulated pPKB; LPL; Ppargc1a mRNA expression	Kuipers et al., 2019 (118)
	C57BL/6 mice	Mice fed HFD and HFrD for 12 weeks	Increased iBAT lipid droplet, and lipid storage pattern resembling WAT	Downregulated VEGF-A, UCP1, B3-AR, Plin1 and Cidea gene expression	Miranda et al., 2020 (119)
	Wistar rats	Mice fed HCD, HFD, HFHSD from 8 weeks to 16 weeks	Increased iBAT adipocyte area more prominent in HFHSD	Downregulated FGF21, PPARγ, SIRT1, CPT1, MAPK3, Gsk3-β, IRS-2, and GLUT4 gene expression	Serdan et al., 2021 (120)
	C57Bl6/J mice	Mice dam fed HFD for 6 weeks prior to mating, and during gestation and lactation	Increased triglycerides, oxidative phosphorylation, and lipid synthesis in BAT of male offspring, while the opposite effect was observed in female	Upregulated Cd36, Cpt1, Plin2, Cidea, and Pparγ mRNA expression and downregulated Ucp1, Dio2, Pgc1α, and Prdm16	Savva et al., 2022 (121)
	C57BL/6J mice	Mice received HFD for 12-, 16- and 20- weeks	Increased large lipid droplets, impaired thermogenesis, increased inflammation, and ER stress, and decreased energy expenditure in iBAT	Upregulated Tlr4, and Nlrp3 mRNA expression, and downregulated UCP1, Bmp8b, Nrg4, Vegfa, Ampk and Sirt-1	Rangel-Azevedo et al., 2022 (14)
Thermoneutral	A/J mice	Mice raised at 22°C or 30°C and fed HFD for 20 weeks	Increased paler brown/beige adipocytes at 30°C, while decreased thermogenic program and sympathetic drive	Downregulated UCP1, PGC1α, Dio2, Elovl3, and Cox1 gene expression, as well as tymine hydroxylase, NETO, and norepinephrine content	Cui et al., 2016 (122)
	C57BL/6J mice	Mice kept at 28°C for 10 days	Increased white-like adipocyte, macrophage infiltration, crown-like structure, and mitochondrial degeneration in iBAT and mBAT	Loss or deficiency of ATGL	Kotzbeck et al., 2018 (13)
	Wild-type mice	Mice housed at 30°C for 3 days, 7 days, or 4 weeks	Induced autophagy, increased white-like unilocular adipocytes, and decreased mitochondrial density in iBAT	Upregulated TFEB gene expression and downregulated UCP1, PGC1α, Cox4i1, Cox7a, and Cox8b	Sass et al., 2021 (123)
Age	C57BL/6 mice	Mice euthanized at 3-, 6-, 9-, or 12-months of age	Increased lipid droplets size and area in classical brown adipocytes while decreased clusters of beige cells and subcutaneous and visceral WAT	Not investigated	Gonçalves et al., 2017 (124)
	Wild-Type and RAG1 KO mice	Mice were used for experiments at 3- and 18-months of age	Inhibited brown adipocyte differentiation, and increased larger unilocular lipid droplets *via* senescent T cells infiltration in iBAT of aged mice	Upregulated IFN-γ gene expression and downregulated UCP1, PPARγ, and PGC1α	Pan et al., 2021 (125)
	Stromal vascular fraction (SVF) cells	SVF cells isolated from BAT were cocultured with senescent T cells	Decreased brown adipocytes differentiation	Downregulated UCP1, PPARγ, PGC1α, PLIN1, and adiponectin gene expression	
	Tianfu Black rabbits	Rabbits were used for experiments at infant stage, 15 days, 85 days, and 2 years	Inhibited brown adipocytes diffferentiation, and increased unilocular adipocytes and decreased multilocular adipocytes in BAT	Increased lncRNAs, and downregulated CYTB, COX2, and ND1 gene expression	Du et al., 2021 (126)
	New Zealand White rabbits	Rabbits were used for experiments at the ages of 1 day and 3, 6, and 12 weeks	Increased brown adipocyte hypertrophy, and restriction of FSTL1+ progenitors adipogenic capacity	Downregulated UCP1 and DIO2 gene expression, linked with FSTL1 deficiency	Haung et al., 2022 (127)
	FVB mice	Mi were used for experiments at 1-, 3-, 6-, and 12- months	Increased unilocular adipocytes in beige adipose tissue while decreased multilocular adipocytes	Not investigated	Scambi et al., 2022 (128)
	Adipose-derived stromal cells	Stromal cells from inguinal WAT of 2-months-old mice	Induced switch from a brown- to white-like precursor transcriptional signature	Upregulated NFkB gene expression and downregulated E2F1	
Genetic mutation	Zucker diabetic fa/fa rats	3 weeks of intervention	Increased large unilocular lipid droplets in iBAT while decreased glucose utilization	Downregulated UCP1	Lapa et al., 2017 (129)
	Leptin receptor-deficient (*db/db*) mice	Mice were used for experiments at 13 weeks of age	Increased white-like unilocular adipocyte, macrophage infiltration, and crown-like structure in iBAT	Not investigated	Kotzbeck et al., 2018 (13)
	Goto-Kakizaki rats	Mice fed HC for 8 weeks	Increased adipocyte area in iBAT while decreased cell density and glucose uptake	Upregulated CPT1, CPT2, SIRT1, PGC1α, and leptin gene expression and downregulated UCP1 and Glut-1	Serdan et al., 2021 (120)

ATGL, adipose triglyceride lipase; AMPK, AMP-activated protein kinase; BAT, brown adipose tissue; BMP8b, bone morphogenetic protein 8 beta; Cd36, cluster of differentiation 36; Cidea, cell death-inducing DNA fragmentation factor-like effector A; CYTB, cytochrome-B; Cpt 1, carnitine palmitoyltransferase 1; Cox, cyclooxygenase; DIO2, iodothyronine deiodinase 2; E2F1, E2F transcription factor 1; ER stress, endoplasmic reticulum stress; FGF21, fibroblast growth factor-21; Fis1, mitochondrial fission 1 protein; FSTL1, follistatin-like 1; GLUT, glucose transporter; Gsk3-β, glycogen synthase kinase 3 β; MAPK3, mitogen-activated protein kinase 3; HC, high cholesterol; HFD, high fat die;, HFHSD, high fat high sugar diet; Hsl, hormone-sensitive lipase; iBAT, interscapular brown adipose tissue; IFN-γ, interferon; LncRNAs, long non-coding RNAs; mBAT, mediastinal BAT; ND1, NADH dehydrogenase 1; NETO, norepinephrine turnover; NRG4, neuregulin 4; IRS-2, insulin receptor substrates 2; Plin, perilipin; PKB, Protein kinase B; PRDM16, PR domain containing 16; PPARγ, peroxisome proliferator-activated receptor-γ; PGC1α/Ppargc1, peroxisome proliferator- activated receptor γ coactivator 1-alpha; Sirt1, Sirtuin 1; Srebp1c, sterol regulatory element-binding protein 1c; TFEB, transcription factor EB; TLR4, Toll-like receptor 4; UCP1, uncoupling protein 1; VEGFs, vascular endothelial growth factors; WAT, white adipose tissue.

#### 3.1.1 High fat diet induced BAT/beige adipose tissue whitening by suppressing angiogenesis and elevating inflammation

It is acknowledged that the consumption of a diet rich in fat and cholesterol, and sugar, is the most common cause of obesity and metabolic syndrome (130, 131). In fact, animal models are a good example of the impact of such a diet, with evidence indicating that feeding rats two individual components of a western-style diet 60% HFD and 55% high fructose diet can cause fat accumulation, drive a state of insulin resistance and other metabolic complications (132–134). Based on growing experimental evidence, exposing rodents to HFD-feeding can cause several pathological abnormalities that include adipocyte hyperplasia and hypertrophy (132). The latter is linked with the state of inflammation, whereby adipose tissue expansion results in the release of proangiogenic cytokines, such as leptin, adiponectin, VEGF, TNF-α, and transforming growth factor beta (TGF-β) angiopoietin; such evidence has been extensively reviewed elsewhere (135, 136). Amongst other cytokines, VEGF-A is a major proangiogenic factor that is commonly downregulated in obesity (137–140). In fact, adipose tissue vascularization regulates chronic inflammation and systemic insulin sensitivity (141). In obese subjects, adipose tissue displays capillary rarefaction and hypoxia, which are paralleled with the infiltration of macrophages and the release of inflammatory cytokines (137, 142, 143). Several lines of evidence showed that HFD and high-fat high-sugar diet induce BAT/beige adipocytes dysfunction and whitening through vascular rarefaction linked with the state of inflammation (116–121).

Although the whitening is less studied in beige adipose tissue compared to BAT, the whitening of beige adipocytes was observed in HFD-fed C57BL/6 mice, and it was marked by enlarged adipocyte size and reduced expression of UCP1 (117). In classical BAT, HFD-feeding progressively induced fat deposition in BAT, which resulted in the expansion and whitening of BAT in female CD-1 mice (64). Similarly, chronic HFD feeding in mice resulted in progressive BAT whitening, which was associated with low energy expenditure, and down-regulation gene expression of Bmp8b, Nrg4, Vegfa involved in regulating vascularization, as well as upregulation of inflammasome and endoplasmic reticulum stress (14). Furthermore, BAT from HFD-fed C57BL/6J mice displayed lipid accumulation and insulin resistance, which were accompanied by reduced glucose and triglyceride-derived fatty acids uptake (118). Of note, Ucp1 gene expression was unaltered, whereas the expression of PGC1α and protein kinase B (PKB) were suppressed, suggesting the impairment of mitochondrial biogenesis and insulin sensitivity (118). More insights on the mechanism showed that HFD-induced iBAT whitening in C57 BL/6 mice, which was accompanied by decreased expression of the genes promoting vascularization, thermogenesis, fatty acids oxidation including VEGF-A, Ucp1, β3-AR, Cidea, and carnitine palmitoyltransferase (CPT) (119). In the same study, they demonstrated that counteracting BAT whitening using PPARα agonists can help to ameliorate the complications associated with obesity (119). The combination of HFD and high-sugar diet impaired BAT function in Wistar rats (120). The evidence of whitening in these animals was marked by the increased adipocytes area and decreased expression of BAT markers, such as FGF21, PPARγ, SIRT1, and CPT1, as well as the genes involved in the insulin signaling including insulin receptor substrate 2 (IRS-2), and glucose transporter (GLUT)-4 (120).

Amid finding the connection between the pathogenesis of obesity and its risk factors, maternal nutrition has become a target to understand the development of obesity and beyond (144). Such nutrition has a significant contribution to the developmental origins of metabolic complications upon growth to adulthood (145). Although adipose tissue plays an utmost important role in newborns as a regulator of energy homeostasis and thermogenesis (146, 147), it is not clear how maternal nutrition affects offspring adipose tissue function. Recently, Savva et al. (121) investigated the impact of maternal HFD on adipose tissue programming in male and female C57Bl6/J mice offspring. Interestingly, only male offspring exhibited a whitening of BAT and impaired metabolic profile whereas female counterparts presented with enhanced thermogenesis and cell differentiation in BAT (121), which can be attributed to the presence of estrogen in females (148). On the other hand, the whitening of BAT in male offspring was accompanied by the upregulation of the genes involved in lipid metabolisms such as Cd36, Cpt1, Cidea, and Pparγ, as well as the downregulation of BAT markers including DIO2, UCP1, PGC1α and PRDM16 (121).

#### 3.1.2 Thermoneutrality-induced BAT whitening through mitophagy stimulation and SNS-derived signals reduction

Ambient temperature has a strong impact on body metabolism and energy expenditure, which in turn affects the morphology and thermogenesis function of BAT. Although the thermoneutral condition of approximately 22°C is the standard living environmental condition for humans, it is known to cause BAT involution and adiposity in rodents (149). However, the effect of seasonal changes on BAT thermogenesis and plasticity is still not well understood in humans. Apparently during winter season, human subcutaneous WAT display increased mRNA expression of UCP1, PGC1α, and transmembrane protein 26 (TMEM26), along with other genes involved in energy utilization and lipolysis, such as adiponectin, acetyl CoA carboxylase (ACC), and HSL (150). However, this effect was suppressed by excessive lipid accumulation and inflammation in obesity (150). In healthy men, whole-body energy expenditure and cold-induced thermogenesis were assessed in both summer and winter, using fluorodeoxyglucose (FDG)-positron emission tomography (PET) combined with computed tomography (CT) (151). Cold-induced thermogenesis was increased in winter compared to summer in a BAT-dependent manner, suggesting that the metabolic activity of human BAT is maximal in winter (151). In terms of glucose metabolism, it was reported that winter swimmers displayed no BAT glucose uptake at a thermal comfort zone while winter swimmers have higher cold-induced thermogenesis than control subjects in young healthy men (152).

In mice, a thermoneutral zone of 30°C is used for thermally humanizing mice BAT, which shows a remarkable resemblance to human BAT (153, 154). It has been reported that thermoneutral housing of mice in conjunction with or without a high-calorie diet, strongly reduces metabolic capacity and increases lipid accumulation in BAT, leading to a “white-like” appearance (13, 122, 123). Indeed, A/J mice housed at 30°C with HFD displayed paler and larger brown or beige adipocytes (122). This was accompanied by reduced SNS and thermogenic program which were evident by decreased tyrosine hydroxylase and norepinephrine turnover, as well as the decreased mRNA expression of UCP1, PGC1α, DIO2, elongation of very long chain fatty acids protein 3 (Elovl3) and cyclooxygenase (Cox)-1 (122). Other evidence showed that BAT whitening can be linked to the recruitment of immune cells involved in pro-inflammation and mitophagy.

An ambient temperature of 28 showed that interscapular and mediastinal BAT from C57Bl/6j mice acquired a white-like unilocular adipocyte phenotype, which involved increased macrophage infiltration, formation of crown-like structures, and degenerating mitochondria, marked by adipose triglyceride lipase (Atgl)-deficiency (13). A study by Sass et al. (123), showed that thermoneutral adaptation at 30 induced BAT whitening which was characterized by increased unilocular adipocytes and mitochondrial degradation in Wild-type mice. This was also accompanied by the decreased gene expression of thermogenic markers, including UCP1, PGC1α, cytochrome c oxidase subunit 4 isoform 1 (Cox4i1), cytochrome c oxidase subunit 7A (Cox7a), and cytochrome c oxidase subunit 8B (Cox8b), while the expression levels of the autophagy-regulating transcription factor EB (TFEB) was continuously increased (123). Moreover, the inhibition of autophagy reversed the whitening in BAT (123). This agrees with the previous evidence indicating that suppression of brown adipocyte autophagy improves energy metabolism in part by regulating mitochondrial turnover in mice (109).

#### 3.1.3 Aging-induced BAT/beige adipose tissue whitening by elevating lncRNAs expression and interferon-γ secretion

Aging has long been implicated in adipose tissue dysfunction and increased risk of obesity (155). A considerable decline in BAT and beige adipose tissue with advancing years and increasing body fat percentage has been determined (156). Generally, aging is closely associated with low-grade systemic inflammation, and alterations in endocrine signals (157); mechanisms that are linked with BAT dysfunction upon aging (156, 158). Several mechanisms that might contribute to the age-related decline in BAT activity have been studied in animals and humans. For instance, Berry et al. (159) have demonstrated that mouse and human beige progenitor cells undergo an age-related senescence-like phenotype that accounts for age-dependent beiging failure; however, genetically or pharmacologically activation of p38/Ink4a-Arf pathway rejuvenated beige progenitors and restored beiging potential. Tajima et al. (160) identified mitochondria lipoylation as a molecular process underlying the age-related decline in BAT thermogenesis of mice, implying that a defect in mitochondrial lipoylation and fuel oxidation in BAT, leads to glucose intolerance and obesity upon aging. Conversely, α-lipoic acid supplementation enhanced mitochondrial lipoylation which in turn restored BAT function in aged mice (160).

A notable observation was made by the decline in BAT content and activity linked with the whitening during adiposity in rodents and rabbits (161). In female C57BL/6 mice aged (6-12 months old), BAT displayed a morphological change toward the fat storage phenotype with increased lipid droplet size and area (124). This was accompanied by the loss of clusters of beige adipocytes from subcutaneous and visceral WAT (124). To elucidate the underlying mechanism, a recent study by Pan et al. (125) reported an increase of unilocular lipid droplets and senescent T cells infiltration which induces BAT whitening *via* interferon (IFN)-γ secretion in 18-month-old and 3-month-old mice. Moreover, IFN-γ lead to the inhibition of brown pre-adipocyte differentiation which contributed to adipose tissue remodeling in aged mice (125). This was further verified using stromal vascular fraction cells isolated from BAT and T cells co-culture, which showed a reduction in UCP1, PPARγ, PGC1α, Plin1, and adiponectin gene expression (125). Although subcutaneous and visceral WAT supposedly comprised of beige adipocytes were enlarged, UCP1 was poorly detected in young mice and it was not detected in old mice, suggesting that there was a complete loss of thermogenic capacity upon aging. A switch from brown/beige- to white-like adipocytes was observed in FVB mice after 12- months of age (128). Moreover, the transcriptional profile of adipose-derived stromal cells mirrors these changes both at mRNA and microRNA transcriptional levels through E2F transcription factor 1 (E2F1) and nuclear factor kappa B (NFkβ) (128).

In 2 years old (aged) rabbits, BAT presented large unilocular lipid droplets with a dramatic decrease in transcriptional copy numbers of the mitochondrial genes, including cytochrome B, COX2, and NADH dehydrogenase 1 (126). Importantly, long non-coding RNAs (lncRNAs) were highly expressed in the BAT of aged rabbits. When assessed using *in vitro* model, these lncRNAs appeared to cause impairment in brown adipocyte differentiation. Presumably, lncRNAs suppressed the expression of brown adipocyte transcriptional factors, however, this requires further investigation (126). Recently, Huang et al. (127) have demonstrated that rabbit BAT involutes in a manner similar to humans with the analogous progenitor hierarchy. A progressively whitening with adipocyte hypertrophy and loss of UCP1 expression in the interscapular, subscapular, and suprascapular BAT depots of the rabbits was readily seen from 3 weeks of age and full conversion to WAT-like tissues at 12 weeks (127). This BAT whitening was associated with the restricted adipogenic capacity of follistatin-like 1 (Fstl1+) progenitors (127). Moreover, deletion of the Fstl1 gene or ablation of Fstl1+ progenitors in mice induced BAT paucity (127). This suggested that lncRNAs can be one of the molecular drivers of BAT whitening upon aging. However, this requires confirmation in human studies.

#### 3.1.4 Genetic models of type 2 diabetes presented BAT whitening

The concept of the genetic alteration as the cause of obesity has been progressively investigated over the past two decades (162). Based upon the years of discoveries, the genetic causes of obesity can be broadly classified into polygenic and monogenic (162). Specifically, polygenic obesity (or common obesity) is caused by multiple gene mutations or polymorphisms that promote weight gain (163). On the other hand, monogenic obesity which is inherited in a Mendelian pattern is typically rare and is characterized by early-onset, high severity, and a single gene mutation in the leptin-melanocortin pathway (162, 163). For these reasons, most people with obesity have certain mutated genes that predispose them to gain excess weight.

To gain a profound understanding of genetic obesity, animal models of obesity and type 2 diabetes such as leptin receptor-deficient *db/db* mice, and Zucker fatty *fa/fa* rats have been widely utilized (161). Over the past few years, these models have been also used to study the impact of genetic obesity on the morphology and function of BAT. However, the special focus herein is on BAT whitening. A study by Lapa et al. (129) showed that BAT from Zucker diabetic fatty *fa/fa* rats, displayed a ubiquitous white adipose-like tissue phenotype, with large unilocular lipid droplets and impaired glucose utilization at 14 weeks of age. This BAT involution was accompanied by the increased synthesis and accumulation of intracellular fatty acids, as well as the decreased expression of UCP1 (129). Although the underlying molecular mechanisms have not been elucidated so far, iBAT from db/db mice displays whitening and crown-like structure formation at 13 weeks of age (13).

In a non-obese model of T2D, which shares a susceptibility locus on chromosome 10 like in humans, lower cell density and higher adipocyte area were recorded in Goto-Kakizaki rats (120). Interestingly, glucose uptake in BAT was impaired in both baseline and even after 30 min of stimulation with 1 mg/kg CL316,243, a β3-adrenergic agonist (120). Subsequently, there was an increased expression of genes involved in fatty acid oxidation (CPT1 and CPT2), BAT metabolism (Sirt1 and PGC1α), but decreased gene expression of GLUT-1 compared to other experimental groups (120). Possibly, impairment of the β3-adrenergic response could suggest an increased expression of the above-mentioned genes, acting as a compensatory mechanism.

### 3.2 Chemically induced BAT/beige adipose tissue whitening

In parallel with other risk factors, chemicals such as endocrine-disrupting chemicals (EDCs) are known to significantly contribute to the high prevalence of obesity (164). These chemicals are found in a wide spectrum of consumer products, like tobacco, flame retardants, and pesticides which people are most likely to be exposed to in their daily life through ingestion, inhalation, or direct dermal contact (164). The potential targets for EDCs are the glucocorticoid and mineralocortcoid receptors, which are members of the steroid receptor subfamily that mediate the actions of glucocorticoids and mineralocorticoids, the main classes of corticosteroids (165). These chemicals can act directly on adipose tissue to induce hypertrophy and dysfunction of this tissue (166). Several lines of evidence have demonstrated that exposure to certain chemicals can negatively impact the phenotype and physiological functions of BAT and beige adipose tissue by inducing whitening (**Table 2**).

**Table 2:** Summary of evidence reporting on BAT/beige adipose tissue whitening induced by chemicals like bevacizumab, nicotine, dechlorane plus, serotonin and glucocorticoid.

Chemical	Model	Intervention protocol	Main findings		Author, year
			Effect	Mechanism	
**Bevacizumab**	C57BL/6 mice	Neonatal mice injected with 1 μg/eye bevacizumab (anti-VEGF antibody) on a postnatal day 14, 17 and 21	Increased lipid droplet expansion in the iBAT while decreased vascular density	Downregulated VEGF levels, and PGC1α and Ucp1 gene expression	Jo et al., 2015 (167)
**Nicotine**	Wistar rat’s offspring	Male offspring from Wistar rats were exposed to 1 mg/kg nicotine twice a day for 26 weeks during pregnancy or lactation	Increased lipid droplet expansion and impaired mitochondria in the iBAT	Downregulated Prdm16, PGC1α, Ucp1 and Cpt2 mRNA expression	Fan et al., 2016 (168)
	Wistar rat’s offspring	Female offspring from Wistar rats were exposed to 1 mg/kg nicotine twice a day for 4- and 26 weeks during pregnancy and lactation	Increased unilocular lipid droplets, impaired angiogenesis, abnormal mitochondria in the iBAT	Downregulated PGC1α, UCP1, Prdm16, Cidea; Vegfr2, Vegf, Hgf, Npy, and Resistin gene expression	Chen et al., 2020 (169)
	C3H10T/12 cells	Differentiated C3H10T/12 cells exposed to 0.5, 5, 50 μM nicotine for 24, 36, and 48 h	Suppressed beige “Brown-like” phenotype and angiogenesis	Downregulated PGC1α, UCP1, Prdm16, Vegf, Hgf, and Ang2 gene expression	
**Dechlorane plus**	C57BL/6 mice	Mice fed HFD and 1000 μg/kg dechlorane plus for 28 days	Increased lipid accumulation and WAT-like phenotype in BAT	Downregulated Ucp1 mRNA expression	Peshdary et al., 2020 (170)
**Serotonin**	HIB1B brown adipocytes	Non-differentiated and differentiated HIB1B brown adipocytes were exposed to 10 μM serotonin with or without palmitic acid for 30 h	Increased transdifferentiation of beige adipocytes into white adipocytes while decreased brown adipocytes differentiation	Upregulated UCP2, FASN, leptin and adiponectin gene expression and downregulated BMP-7, UCP1, FGF21, pAMPK, Prdm16 and Pparγ, and Cpt1	Rozenblit-Susan et al., 2018 (171)
**Glucocorticoid**	CD1 mice	Mice were orchidectomized or ovariectomized prior to exposure to 250 μg/day of corticosterone for 4 weeks, with or without androgen	Increased androgens sensitized glucocorticoid-induced intracellular lipid accumulation and lipid droplet size expansion in the BAT	Downregulated UCP1 gene expression while Ppargc1a, Pparg, and Acaca remain unaltered	Gasparini et al., 2019 (172)
	C57BL/6J wild-type mice	Mice injected with 5 mg/kg dexamethasone every second day for 1 week	Increased autophagy, enlarged lipid droplets, and triglycerides in the iBAT	Upregulated ATG7, BTG1, Rb1, Nrip1, Rbl1/p107 gene expression, and downregulated UCP1	Deng et al., 2020 (173)
	Brown adipocytes precursor cells	Precursor cells were isolated from scapular fat of newborn Wild-Type mice, differentiated, and exposed to 1 µM dexamethasone for 24 h	Increased fat mass and autophagy, and decreased oxygen consumption rate	Upregulated ATG7 and BTG1 gene expression, and downregulated Ucp1, Nrip1 and Agt mRNA expression	
	C57BL/6 (C57BL/6NCrl) mice	Male mice were exposed to oral treatments of 50 µg/ml corticosterone for 4 weeks	Increased adipocyte area, insulin resistance, and weight of the iBAT, while mitochondrial content remain unchanged	Downregulated UCP1 gene expression	Bel et al., 2022 (174)

Acaca, acetyl-coa carboxylase alpha; Agt, angiotensinogen; Ang2, angiopoietin-2; AMPK, AMP-activated protein kinase; ATG7, autophagy-related 7; iBAT, interscapular brown adipose tissue; BTG1, B cell translocation gene 1; Bmp7, bone morphogenetic protein 7; Cidea, cell death-inducing DNA fragmentation factor-like effector A; Cpt 1, carnitine palmitoyltransferase 1; FGF21, fibroblast growth factor-21; Fis1, mitochondrial fission 1 protein; Hgf, hepatocyte growth factor; HFD, high fat diet; Npy, neuropeptide; Nrip1, nuclear receptor interacting protein 1; PRDM16, PR domain containing 16; PPARγ, peroxisome proliferator-activated receptor gamma; PGC1α, peroxisome proliferator-activated receptor γ coactivator 1-alpha; Rb1, RB transcriptional corepressor 1; Rbl1/p107, RB transcriptional corepressor like 1; UCP1, uncoupling protein 1; VEGFs, vascular endothelial growth factors; WAT, white adipose tissue.

#### 3.2.1 Bevacizumab impaired vascular network and induced whitening in BAT

Bevacizumab, a recombinant humanized anti-vascular endothelial growth factor (VEGF) antibody, is trailed in retinopathy of premature infants (175). Although anti-VEGF agents are the first-line treatment for various angiogenesis-related retinal diseases, it is not clear how the anti-VEGF antibody can accelerate the risks of systemic complications after intravitreal injection in premature infants (176). A study by Jo et al. (167) showed that intravitreally injection with anti-VEGF antibody (1 μg/eye) increases lipid droplet accumulation and induces the loss of vascular network in neonatal C57BL/6 mice. In addition to reduced VEGF levels, this was accompanied by the downregulation of mitochondria-related genes PGC1α and UCP1 (167). Since BAT is a highly vascularized tissue, it is evident that anti-VEGF agents interfere with BAT vascularization, which in turn induces BAT whitening and dysfunction.

#### 3.2.2 Nicotine exposure during the prenatal and lactation period induces BAT/beige adipose tissue whitening in offspring

Nicotine is a chemical that is widely found in tobacco, and it has been associated with many health problems (177). Accordingly, epidemiological studies have reported that maternal smoking during pregnancy might be a serious risk factor for childhood obesity (178). Of major concern, maternal nicotine exposure has become a growing risk factor for the health of the offspring and the origin of chronic diseases beyond infancy. For example, prenatal or lactation exposure to nicotine is associated with increased gonadal and inguinal subcutaneous WAT depots and dysfunction in offspring (179). However, the impact of nicotine on BAT structure and function is not well understood. Previously, it was reported that nicotine exposure in adult mice increases BAT thermogenesis and promotes weight loss *via* the inactivation of hypothalamic AMPK (180), and induces WAT browning through the hypothalamic κ opioid receptor (181). Paradoxically, maternal nicotine exposure induces BAT dysfunction and weight gain in both male offspring (168, 169).

In male rat offspring exposed to 1 mg/kg nicotine during pregnancy and lactation, BAT displayed a whitening phenotype characterized by lipid droplet accumulation and impaired mitochondrial structure (168). Moreover, the expression of BAT structure and function-related genes such as PRDM16, PGC1α, UCP1, and CPT2 was decreased. Similarly (169), female rat offspring exposed to 1 mg/kg nicotine during pregnancy and lactation presented white-like adipocytes, impaired angiogenesis, and abnormal mitochondrial structure in iBAT. This was accompanied by a down-regulation of brown-like genes PGC1α, UCP1, Prdm16, and Cidea, as well as a decrease in BAT secretion of pro-angiogenic factors including VEGF, VEGF receptor 2, hepatocyte growth factor (Hgf), neuropeptide (Npy), and resistin (169). This was further confirmed *in vitro* using C3H10T1/2 cells, showing a reduction of beige “brown-like” phenotype and angiogenesis, as well as brown-like gene expression of PGC1α and UCP1. Altogether, this evidence suggests that nicotine disrupts angiogenesis in the early development stage and impairs blood vessel formation to induce BAT whitening through downregulation of the PGC1α–UCP1 signals.

#### 3.2.3 Dechlorane plus disrupted mitochondrial UCP1 and induced BAT whitening

Dechlorane plus, an endocrine-disrupting chemical found in flame retardants, is a potential obesogen (182). Apparently, dechlorane plus can induce adiposity by promoting adipogenesis in cultured adipocytes *via* PPARγ independent mechanism (183). Consistently, Peshdary et al. (170) reported that 1000 μg/kg dechlorane plus induces a WAT-like phenotype and disrupts the function of BAT in part by downregulation of UCP1 mRNA expression in C57BL/6 mice. These outcomes are in line with UCP1 gene knockout in mice suggesting that loss of UCP1 could result in the whitening of BAT (184, 185). However, more research on the impact of dechlorane plus on BAT and beige adipose tissue function is warranted to better understand its influence on obesity and other related diseases.

#### 3.2.4 Serotonin-impaired brown adipocytes differentiation and induced beige adipocyte whitening

Endogenous chemicals such as neurotransmitters like serotonin are known to regulate adipogenesis (186). A study by Rozenblit-Susan et al. (171) demonstrated that serotonin (10 μM) induces whitening in palmitic acid-exposed HIB1B adipocytes by shifting their metabolism to lipogenesis rather than lipid oxidation in part by, suppressing brown adipocytes differentiation and inducing beige adipocytes transdifferentiation into white adipocytes. Moreover, this was confirmed by the downregulation of brown adipocyte differentiation markers, such as Prdm16, Bmp7, and Pparγ (171). Consistently, expression of BAT markers such as UCP1 and FGF21, as well as pAMPK/AMPK ratio were downregulated while genes regulating lipogenesis fatty acid synthase (FASN), leptin, and adiponectin were upregulated (171). The observed effects of serotonin require further investigations in human adipose tissue, supposedly similar effects attained in humans could have major implications on obesity reducing WAT and increasing BAT activity.

#### 3.2.5 Glucocorticoids induced BAT whitening by stimulating autophagy

The class of steroid hormones glucocorticoids and corticosterone are known to modulate glucose homeostasis in humans and rodents, respectively (187). Hypercortisolism caused by either endogenous overproduction of glucocorticoids or exogenous administration of glucocorticoids as anti-inflammatory medication can induce the development of obesity. A study by Gasparini et al. (172) reported that corticosterone (250 μg/day) induces intracellular lipid accumulation and reduces UCP1 expression in BAT of CD1 mice. Interestingly, no significant changes were observed in PGC1α, FASN, or acetyl-coa carboxylase alpha (Acaca) expression, implying that changes in lipid accumulation did not directly involve mitochondrial biogenesis, adipogenesis, or lipogenesis in BAT (172).

Several potent synthetic glucocorticoids including dexamethasone have been developed for pharmacological use (187). For example, dexamethasone has been implicated in the study of adipogenesis (188). Here, Deng et al. (173) showed that exposing C57BL/6 mice to 5 mg/kg dexamethasone for one week induced autophagy and lipid droplet expansion in iBAT. Similar outcomes were observed *in vitro* using brown adipocytes precursor cells. Notably, dexamethasone increased ATG7 expression, in part by increasing the expression of B cell translocation gene 1 (BTG1) that stimulates the activity of CREB1 (173). Consistently, UCP1 expression was downregulated, while the expression of WAT marker genes RB transcriptional corepressor 1 (Rb1), nuclear-receptor-interacting protein 1 (Nrip1), and Rbl1/p107 (RB transcriptional corepressor like 1) were upregulated (173). Other evidence showed that chronic exposure to corticosterone can induce the whitening of BAT in C57BL/6 mice, this was evident by increased adipocyte area and elevated expressions of UCP1 in BAT (174). Of note, the whitened phenotype has not been previously associated with increased uncoupling proteins under chronic stress, however, Bel et al. (174) suggested that the increased UCP1 expression could be a compensatory mechanism under certain stress.

## 4 Summary and outlook

**Figure 4:**
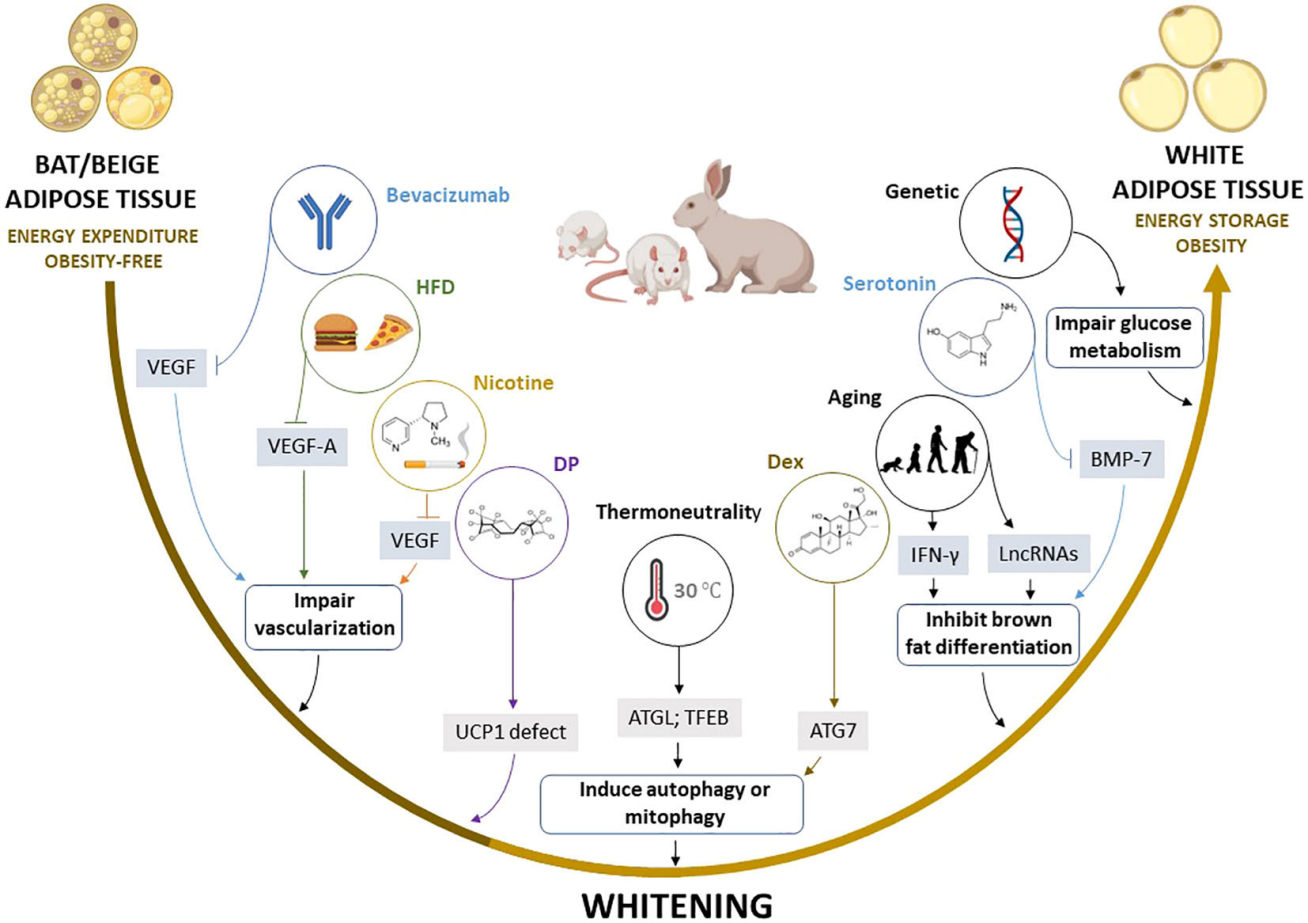
Summary of the proposed mechanisms by which multiple factors induce the whitening of brown (BAT) and beige adipose tissue. Factors such as high fat diet (HFD), bevacizumab, and nicotine induce the whitening by suppressing pro-angiogenic cytokines such as VEGF and VEGF-A, which inhibit angiogenesis and impair vascularization in BAT and beige adipose tissue, whereas thermoneutral zone and dexamethasone (Dex) exposure increase ATG7 and TFEB, which stimulate autophagy or mitophagy. Other factors such as aging, and elevated serotonin induce BAT/beige adipose tissue whitening by increasing INF-γ, and LncRNAs, and decreasing BMP-7, which in turn inhibits brown adipocyte differentiation. Moreover, dechlorane plus (DP) contributes to whitening in part by inhibiting UCP1 expression. ATG7, autophagy-related 7; LncRNAs, long non-coding RNAs; BMP-7, bone morphogenetic protein 7; BTG1, B cell translocation gene 1; IFN-γ, interferon-gamma; UCP1, uncoupling protein 1; TFEB, transcription factor EB; VEGF, vascular endothelial growth factor.

Although the remodeling of BAT and beige adipose tissue through whitening appears to be more common in obesity, it remains unclear how this maladaptive process occurs. Several lines of evidence have demonstrated that the BAT/beige adipose tissue whitening is multifactorial in origin. Indeed, various factors such as diet, age, genetics, thermoneutrality, and chemical exposure have been shown to greatly influence the whitening of adipose tissue by targeting different mechanisms (**Figure 4**). Apart from these factors, chronic exposure to high levels of particulate matter, a complex mixture of solid and liquid particles derived from human activities and natural sources, also promotes the whitening of BAT, as reviewed by Guardia and Shin (110). Although the activity of BAT has been strongly linked with the protection against obesity, fatty liver, and T2D (189), the dysfunction or whitening of BAT in obesity, may contribute to or exacerbate other metabolic complications (190). For instance, recent evidence has demonstrated that severe hyperprolactinemia can also promote BAT whitening and aggravates HFD-induced metabolic dysregulations (191). This suggests that the whitening could represent one of the complications implicated in the pathogenesis of obesity, and it can lead to other secondary complications.

Of note, experimental evidence discussed in this review indicates that autophagy, inflammation, and impairment of angiogenesis “vascularization” are central processes implicated in BAT/beige adipose tissue whitening (83, 108, 167, 169). For example, the most prominent mechanisms implicated in adipose tissue whitening include the inhibition of VEGFs, PGC1α, and BMP-7 which impair vascularization, mitochondrial biogenesis, and brown adipocyte differentiation, respectively. This may lead to more sophisticated processes like infiltration of INF-γ secreting T cells, increased autophagy, and impaired substrate metabolism. Such evidence is in line with the finding from gene knockout models, which suggested that the whitening of BAT is under the control of β-AR (13), BMP (192), and mitochondrial transcription factor A (193), and other genes regulating BAT function (194–198), as well as miRNAs that regulate multiple processes including the differentiation and function of brown adipocytes (199, 200). Based on the current evidence, there are several potential marker genes that are involved in regulating BAT/beige adipose tissue whitening (**Table 3**). However, this requires further investigations in both non-clinical and clinical settings. Particular attention should be placed on identifying plausible therapeutic avenues to prevent or reverse adipose tissue whitening in obesity. This includes assessing and understanding the therapeutic effects of prominent agents like metformin in targeting the adipose tissue to manage obesity-associated complications (201).

**Table 3:** An overview of the potential biomarkers of BAT/beige adipose tissue whitening.

Gene	Upregulation/Downregulation (knockout)	Association with or implication in whitening	Author, year
ATGL	Downregulation	Induce mitophagy, brown adipocyte death, and crown-like structure formation	Kotzbeck et al., 2018 (13)
ATG7	Upregulation	Induce autophagy and increase adiposity	Deng et al., 2020 (173)
BTG1	Upregulation	Induce autophagy and increase adiposity	
BMP-7	Downregulation	Inhibit brown adipocytes differentiation	Rozenblit-Susan et al., 2018 (171)
PRDM16	Downregulation	Inhibit brown adipocytes differentiation	
IFN-γ	Upregulation	Inhibit differentiation of preadipocyte brown adipocytes	Pan et al., 2021 (125)
FSTL1	Downregulation	Loss of brown adipogenic competence of progenitors	Haung et al., 2022 (127)
LncRNAs	Upregulation	Impair brown adipocyte differentiation	Du et al., 2021 (126)
TFEB	Upregulation	Induce mitophagic mitochondrial degradation *via* the autophagosomal and lysosomal machinery	Sass et al., 2021 (123)
UCP1	Downregulation	Increase glucose intolerance, large unilocular adipocytes, and inflammation markers, and decrease mitochondrial subunit protein	Winn et al., 2017 (184); Peshdary et al., 2020 (170)
VEGF	Downregulation	Suppress angiogenesis and decrease vascular density	Jo et al., 2015 (167); Chen et al., 2020 (169)
VEGF-A	Downregulation	Increase inflammation and endoplasmic reticulum stress, and decrease anti-inflammatory cytokines	Miranda et al., 2020 (119); Rangel-Azevedo et al., 2022 (14)

ATGL, adipose triglyceride lipase; ATG7, autophagy-related 7; BMP-7, bone morphogenetic protein 7; BTG1, B cell translocation gene 1; FSTL1, follistatin-like 1; IFN-γ, interferon gamma; LncRNAs, long non-coding RNAs; PRDM16, PR domain containing 16; UCP1, uncoupling protein 1; TFEB, transcription factor EB; VEGF, vascular endothelial growth factor.

## Author contributions

KZ, PVD, and SM-M- concept and original draft. KZ and SXM performed the literature search, study selection, and data extraction. KZ, PD, SXHM, BBN, SEM, BUJ, TMN and SEM-M - manuscript writing and approval of the final draft. All authors contributed to the article and approved the submitted version.

## Glossary

**Table:**

**Acaca**	acetyl-coa carboxylase alpha
**AMPK**	AMP-activated protein kinase
**ATG7**	autophagy related 7
**BAT**	brown adipose tissue
**β3-AR**	Beta-3 adrenegic receptor
**BTG1**	B cell translocation gene 1
**Bmp7**	bone morphogenetic protein 7
**BMP8b**	bone morphogenetic protein 8 beta
**Cd36**	cluster of differentiation 36
**Cidea**	cell death-inducing DNA fragmentation factor-like effector A
**CPTs**	carnitine palmitoyltransferases
**Cox**	cyclooxygenase
**Dex**	dexamethasone
**Dio2**	iodothyronine deiodinase 2
**DP**	dechlorane plus
**EPDR1**	ependymin-related protein 1
**EDCs**	endocrine-disrupting chemical
**Elovl3**	Elongation of very long chain fatty acids protein 3
**FGF21**	fibroblast growth factor-21
**Fis1**	mitochondrial fission 1 protein
**GLUT**	Glucose transporter
**Gsk3-β**	glycogen synthase kinase 3 β
**Hgf**	hepatocyte growth factor
**HFD**	high fat diet
**HFHSD**	high fat high sugar diet
**HSL**	hormone-sensitive lipase
**IL-6**	interleukin-6
**IGF-1**	insulin-like growth Factor-1
**IGFBP-2**	Insulin-like growth factor-binding protein 2
**IFN-γ**	interferon
**lncRNAs**	long non-coding RNAs
**IRS-2**	insulin receptor substrates 2
**MAPK3**	mitogen-activated protein kinase 3
**NGF**	nerve-growth factor
**NRG4**	neuregulin 4
**Npy**	neuropeptide
**Nrip1**	nuclear receptor interacting protein 1
**NETO**	norepinephrine turnover
**Plin**	perilipin
**PKB**	Protein kinase B
**PRDM16**	PR domain containing 16
**PPARγ**	peroxisome proliferator-activated receptor-γ
**PGC1α**	peroxisome proliferator- activated receptor γ coactivator 1-alpha
**Rb1**	RB transcriptional corepressor 1
**Rbl1/p107**	RB transcriptional corepressor like 1
**Sirt1**	Sirtuin 1
**Srebp1c**	sterol regulatory element-binding protein 1c
**TFEB**	transcription factor EB
**UCP1**	uncoupling protein 1
**VEGFs**	vascular endothelial growth factors; 12,13-dihydroxy-9Z-octadecenoic acid
